# Normal breathing releases SARS-CoV-2 into the air

**DOI:** 10.1099/jmm.0.001328

**Published:** 2021-02-25

**Authors:** Piero Di Carlo, Katia Falasca, Claudio Ucciferri, Bruna Sinjari, Eleonora Aruffo, Ivana Antonucci, Alessandra Di Serafino, Arianna Pompilio, Verena Damiani, Domitilla Mandatori, Simone De Fabritiis, Beatrice Dufrusine, Emily Capone, Piero Chiacchiaretta, William H. Brune, Giovanni Di Bonaventura, Jacopo Vecchiet

**Affiliations:** ^1^​ University "G. d'Annunzio" of Chieti-Pescara, Department of Advanced Technologies in Medicine & Dentistry, Chieti, Italy; ^2^​ Center for Advanced Studies and Technology (CAST), University "G. d'Annunzio" of Chieti-Pescara, Chieti, Italy; ^3^​ University "G. d'Annunzio" of Chieti-Pescara, Department of Medicine and Aging Sciences, Chieti, Italy; ^4^​ Clinic of Infectious Diseases, S.S. Annunziata Hospital, Chieti, Italy; ^5^​ Department of Psychological, Health and Territorial Sciences, University "G. d'Annunziio" of Chieti_Pescara, Chieti, Italy; ^6^​ University "G. d'Annunzio" of Chieti-Pescara, Department of Medical, Oral and Biotechnological Sciences, Chieti, Italy; ^7^​ University "G. d'Annunzio" of Chieti-Pescara, Department of Neuroscience, Imaging and Clinical Sciences, Chieti, Italy; ^8^​ Pennsylvania State University, Department Meteorology and Atmospheric Science, University Park, PA, 16802, USA

**Keywords:** Aerosol, CoViD-19, Infection, RT-PCR, SARS-CoV-2 RNA, Viral RNA

## Abstract

This study tests the release of SARS-CoV-2 RNA into the air during normal breathing, without any sign of possible risk of contagion such as coughing, sneezing or talking. Five patients underwent oropharyngeal, nasopharyngeal and salivary swabs for real-time reverse transcriptase PCR (RT-PCR) detection of SARS-CoV-2 RNA. Direct SARS-CoV-2 release during normal breathing was also investigated by RT-PCR in air samples collected using a microbiological sampler. Viral RNA was detected in air at 1 cm from the mouth of patients whose oropharyngeal, nasopharyngeal and salivary swabs tested positive for SARS-CoV-2 RNA. In contrast, the viral RNA was not identified in the exhaled air from patients with oropharyngeal, nasopharyngeal and salivary swabs that tested negative. Contagion of SARS-CoV-2 is possible by being very close to the mouth of someone who is infected, asymptomatic and simply breathing.

## Introduction

The ability of SARS-CoV-2 to infect people is well recognized through droplets from coughing, sneezing, talking or by contact with contaminated matters [1, 2]. On the other hand, airborne transmission during breathing is mostly controversial [3]. Here, we evaluated the presence of SARS-CoV-2 RNA in the exhaled air of symptomatic patients and in air samples collected inside a hospital isolation room.

## Methods and Results

From 21 April to 16 June 2020, five patients were admitted at the Clinic of Infectious Diseases of the ‘SS Annunziata’ Hospital of Chieti (Italy) in a negatively pressurized room, where oropharyngeal, nasopharyngeal and salivary swabs were administered to test for CoViD-19 [4]. To understand if the SARS-CoV-2 RNA was diffused in the ambient air, the isolation room was sampled continuously using a Sartorius Airscan microbiological sampler (Sartorius AG, Gottingen, Germany) with a gelatin membrane filter (80 mm diameter) at 50 l min^−1^. The sampler ran continuously, whereas the filter was replaced every 24 h, just before routine cleaning of the room, and was analysed for the presence of the SARS-CoV-2 RNA.

Direct SARS-CoV-2 RNA release during normal breathing was also investigated by using another microbiological sampler (portable Sartorius AirPort) with a gelatin membrane filter (80 mm diameter) at a flow rate of 50 l min^−1^. Measurements were carried out for 30 min for each distance (1, 10 and 100 cm) from the patient's mouth, and a gelatin membrane filter was changed at each distance to be analysed for the presence of the SARS-CoV-2 RNA. Moreover, the air sampling as a function of the distance from the patient's mount was carried out under diverse conditions (with/without surgical mask) to better understand how the viral RNA spreads and the efficacy of the personal protective equipment.

Specific real-time reverse transcriptase PCR (RT-PCR) (TaqMan 2019-nCoV Assay Kit v2; Thermo Fisher Scientific, Italy) targeting RNA-dependent RNA polymerase was used to detect the presence of SARS-CoV-2 RNA through: (a) ORF1ab, N gene and S gene and (b) number of cycles required for the fluorescent signal to cross the threshold in rRT-PCR, to quantify the viral load, which is a relative measure of the amount of viral RNA in the air [4]. In detail, each single-tube kit besides the three assays that target SARS-CoV-2 genes (ORF1ab, N protein, S protein), includes one internal positive control (IPC) that targets exogenous control RNA from bacteriophage MS2. Each assay is linked to a different dye: ORF1ab to 6-FAMTM, N protein to VICTM, S protein to ABYTM, MS2 IPC to JUNTM. Moreover, the assays are designed to use nucleic acid, that was isolated through the MagMAXTM Viral/Pathogen Nucleic Acid Isolation Kit (Themo Fisher Scientific, Milan, Italy) [4].

Data were analysed through the dedicated software ‘Design and Analysis’ (Thermo Fisher Scientific). The settings were indicated by Thermo Fisher Scientific (Run Mode: Standard, Passive Reference: None, Threshold: 5000, Baseline: 5 and cut-off: 37). The results were interpreted according to the manufacturer manual: if 2/3 or 3/3 assays were positive, then the SARS-CoV-2 RNA was considered present, whereas if 1/3 or none of the assays was positive, then the SARS-CoV-2 RNA was considered absent. To be considered as ‘positive’, the Ct value of 2/3 or 3/3 assays had to be <37. If the Ct value obtained was 37 to 40, the test was repeated.

During the 7-week study period, five patients (mean age: 75.4 years old) infected with SARS-CoV-2 with bilateral interstitial pneumonia and hyper-inflammation (defined as CRP ≥40 mg l^−1^) were hospitalized in a double room since they showed the same health status; however, during the tests reported here there was always one patient in a room. Here, the Sartorius Airscan microbiological sampler was positioned on a table 1 m from the patient’s bed to collect ambient room airborne samples throughout 6 days, although the gelatin membrane filter was changed every 24 h to have daily samples to be analysed for the presence of the SARS-CoV-2 RNA. Moreover, by using the portable Sartorius AirPort instrument, samples were collected from the patients’ mouths as a function of distance (1, 10 and 100 cm) for 30 min at each distance and for each distance the gelatin membrane filter was replaced to be analysed with the RT-PCR. The room was cleaned with sodium hypochlorite 0.1 % and ethanol 90%, three times per day, while the room air exchanged was every 72 h.

Patient 1 was admitted to the hospital for other symptoms (not for CoViD-19), but after 21 days of hospitalization the CoViD-19 symptoms appeared and, after 7 days of CoViD-19 symptoms, oropharyngeal, nasopharyngeal and salivary swabs were taken, along with expired air sampled at both 1 and 100 cm of distance from the patient’s mouth.

Patient 2 was admitted to the hospital for other symptoms (not for CoViD-19), but after 19 days of hospitalization the CoViD-19 symptoms appeared and, after 9 days of CoViD-19 symptoms, besides oropharyngeal, nasopharyngeal and salivary swabs and the air samples collected at 1 and 100 cm away from the mouth, one more sample was collected 1 cm away from the mouth while the patient was wearing a surgical mask.

Patient 3 was admitted to the hospital for other symptoms (not for CoViD-19), but after 28 days of hospitalization the CoViD-19 symptoms appeared and, after 3 weeks of CoViD-19 symptoms was still infected, based on three consecutive positive swab samples. Therefore, oropharyngeal, nasopharyngeal and salivary swabs were taken, along with air samples collected at both 1 and 100 cm away from the mouth of the patient normally breathing and without a surgical mask.

Patient 4 was admitted to the hospital for other symptoms (not for CoViD-19), but after 45 days of hospitalization the CoViD-19 symptoms appeared and, after 6 weeks of CoViD-19 symptoms, was still infected based on six consecutive positive swab samples. Therefore, nasopharyngeal and salivary swabs were taken, along with air samples collected at 1, 10 and 100 cm away from the mouth of the patient normally breathing, both with and without a surgical mask.

Patient 5 was admitted to the hospital for other symptoms (not for CoViD-19), but after 59 days of hospitalization the CoViD-19 symptoms appeared and, after 8 weeks of CoViD-19 symptoms was still infected based on five consecutive positive swab samples. Therefore, nasopharyngeal and salivary swabs were taken, along with air samples collected at 1 and 100 cm away from the mouth of the patient normally breathing, both with and without surgical mask.

The SARS-CoV-2 RNA was detected in air at 1 cm from the mouth of symptomatic patients whose oropharyngeal, nasopharyngeal and salivary swabs tested positive (Table 1). The patients, during the air sampling, never coughed, sneezed, nor talked, but breathed weakly due to their clinical conditions, worsened further due to their old age. On the other hand, SARS-CoV-2 RNA was not detected in air at all distances from the mouth on patients tested negative at oropharyngeal, nasopharyngeal and salivary swabs (Table 1). The negative samples collected both at 100 cm distance from the mouth and at 1 cm when the patient was wearing a mask confirm that social distancing, along with a correct and consistent use of face-masks, represent effective measures in preventing interpersonal infection by SARS-CoV-2 RNA [5]. In fact, in patient 2, despite the high viral load (ORF1ab=25. 573 cycles; N gene=25.584 cycles; S gene=25.411) showed in the air sample collected 1 cm away from the mouth, the measurements at the same distance in the presence of a surgical mask changed completely the result, turning it from positive to negative (Table 1). However due to the limited number of samples with patients wearing a surgical mask, more investigations are needed to confirm these results.

**Table 1.** Assessment of SARS-CoV-2 RNA presence in swabs (oropharyngeal, nasopharyngeal, salivary) and ambient air samples. All samples were assayed for SARS-CoV-2 RNA using RT-PCR, through the detection of ORF1ab, N gene and S gene. Cycle threshold value is referred to the number of cycles necessary for the fluorescent signal to cross the threshold, considering threshold=5.000, baseline=5, and cut-off=37 cycles. The viral load was considered higher as long as the cycle threshold value was lower. Samples were considered ‘Positive’ when at least two genes have a cycle threshold value <37, whereas ‘Negative’ when the cycle threshold value is Undetermined or >37. All air samples collected in the patients’ room, starting on the day of the observation, were negative.

	RT-PCR swab analysis on the day of the air sample		Air sample RT-PCR analysis		
	**Target**	**Cycle threshold value**	**Distance away from the patient’s mouth**	**Target**	**Cycle threshold value**
**Patient 1** **Symptoms:** Cough, fever, shortness of breath					
Oropharyngeal: **Positive**	ORF1ab	26.331	1 cm: **Positive**	ORF1ab	32.764
	N	26.740		N	33.404
	S	26.189		S	33.239
Nasopharyngeal: **Positive**	ORF1ab	27.886	100 cm: **Negative**	ORF1ab	36.535
	N	27.035		N	U*
	S	28.563		S	39.877
Salivary: **Positive**	ORF1ab	29.749			
	N	29.883			
	S	26.846			
**Patient 2** **Symptoms:** Cough, fever, shortness of breath					
Oropharyngeal: **Positive**	ORF1ab	13.787	1 cm: **Positive**	ORF1ab	25.573
	N	15.710		N	25.584
	S	13.589		S	25.411
Nasopharyngeal: **Positive**	ORF1ab	15.221	100 cm: **Negative**	ORF1ab	U*
	N	16.871		N	U*
	S	14.324		S	U*
Salivary: **Positive**	ORF1ab	23.474	1 cm†: **Negative**	ORF1ab	U*
	N	28.971		N	U*
	S	28.372		S	U*
**Patient 3** **Symptoms:** Cough, fever, shortness of breath					
Oropharyngeal: **Negative**	ORF1ab	U*	1 cm: **Negative**	ORF1ab	U*
	N	U*		N	U*
	S	U*		S	U*
Nasopharyngeal: **Negative**	ORF1ab	U*	100 cm: **Negative**	ORF1ab	U*
	N	U*		N	U*
	S	U*		S	U*
Salivary: **Negative**	ORF1ab	U*			
	N	U*			
	S	U*			
**Patient 4** **Symptoms:** Cough					
Oropharyngeal: **Negative**	ORF1ab	U*	1 cm: **Negative** 10 cm: **Negative**	ORF1ab	U*
	N	U*		N	U*
	S	U*		S	U*
Nasopharyngeal: **Negative**	ORF1ab	U*	100 cm: **Negative**	ORF1ab	U*
	N	U*		N	U*
	S	U*		S	U*
Salivary: **Negative**	ORF1ab	U*	1 cm†: **Negative**	ORF1ab	U*
	N	U*		N	U*
	S	U*		S	U*
**Patient 5** **Symptoms:** Astenia					
Oropharyngeal: **Positive**	ORF1ab	U*	1 cm: **Negative**	ORF1ab	U*
	N	U*		N	U*
	S	U*		S	U*
Nasopharyngeal: **Positive**	ORF1ab	U*	100 cm: **Negative**	ORF1ab	U*
	N	U*		N	U*
	S	U*		S	U*
Salivary: **Negative**	ORF1ab	U*	1 cm†: **Negative**	ORF1ab	U*
	N	U*		N	U*
	S	U*		S	U*

*U, Undetermined.

†Air sample collected while the patient was wearing a surgical mask.

## Discussion

Coronaviruses utilize their S protein to enter human cells via surface enzyme angiotensin-converting enzyme 2 (ACE2) receptor [6]. The acute respiratory distress syndrome in CoViD-19 has been marked by upregulation of proinflammatory cytokines and chemokines [7]. SARS-CoV-2 infection causes fever, cough and myalgias. Mild disease may resolve without medical care or may progress to pneumonia and respiratory failure requiring hospitalization [8]. The correct knowledge of the spread of the viral RNA is crucial to avoid mortality and morbidity related to this infection.

Contagion of SARS-CoV-2 is possible by being very close to the mouth of an infected person who may be asymptomatic, is not releasing small droplets by coughing, and is only breathing. Thus, contagion is possible for situations in which social distancing guidelines are difficult to follow, such as for medical or dental examinations, or are ignored, such as in crowded environments or interpersonal relationships. Moreover, the air sampled in the room was negative for SARS-CoV-2 RNA, suggesting that the negative pressurized equipment rooms in hospitals are safer environments for healthcare workers and a good prevention practice to keep the viral RNA from spreading.

In conclusion, social distancing, wearing a face mask [9], and limiting or avoiding personal contact, such as hugging and greeting by kissing, are even more vital in limiting the spread that previously thought. Further studies are required to confirm these results, which have limitations. First, sample size is small due to operational limitations. Second, because the 24 h ambient air filters sampled 72 m^3^ of air, which is 1/3 of the room volume, while the room air was exchanged every 72 h, the amount of SARS-CoV-2 RNA that would have been present in the room with static air flow was diluted. Despite these limitations, these results add to a growing number of studies showing that even breathing can spread SARS-CoV-2 RNA.

### Data availability statement

The data sets used and analysed during the current study are available from the corresponding author upon request.
